# Volume mismatch indicates tumors in paramedial bithalamic diseases: a retrospective study

**DOI:** 10.3389/fneur.2023.1154823

**Published:** 2023-07-25

**Authors:** Lu Su, Peiyi Gao

**Affiliations:** ^1^Beijing Tiantan Hospital, Capital Medical University, Beijing, China; ^2^Neuroradiology Center of Beijing, Neurosurgical Institute, Beijing, China

**Keywords:** paramedial bilateral thalamus, volume mismatch, discriminating, tumors, non-tumors

## Abstract

**Objective:**

This study aimed to investigate the diagnostic performance of volume mismatch sign on discriminating paramedial bithalamic tumors from non-tumors.

**Methods:**

In this study, we recruited patients with tumors or non-tumors of the paramedial bithalamus. We confirmed the diagnosis by pathology, laboratory tests documented in medical records, medical imaging at the baseline, or through at least 1 year of follow-up. Cases with paramedial thalamic lesions on only one side or purely midbrain illnesses were excluded. Additionally, any case without involvement of the medial thalami (i.e., those with one or both-sided anterior, lateral, or posterior thalamic lesions) was excluded. Two neuroradiologists were trained independently to evaluate volume mismatch sign on magnetic resonance T2-weighted images or T2 fluid-attenuated inversion recovery images. A positive volume mismatch sign means that the ratio of the larger-sided lesion volume to the smaller-sided lesion volume is >150%. The volume of each lesion was calculated by multiplying the anteroposterior diameter by the left-right diameter and by the height of the lesion and then dividing by 2. The kappa value was calculated to show the consistency between the two observers. The chi-square test was used to evaluate differences in volume mismatch sign between the bilthalamic midline tumor and non-tumor groups. The positive (PPV) and negative (NPV) predictive values, sensitivity, and specificity were calculated to evaluate the ability of volume mismatch sign to differentiate paramedial bilateral thalamus tumors from non-tumors. A two-tailed *P* ≤ 0.05 was considered to be statistically significant. The analyses were performed using the statistical software SPSS version 26.

**Results:**

A total of 96 patients were enrolled in this study between March 2012 and October 2022. A high agreement between the two observers on the volume mismatch sign of bilateral paramedian thalamic diseases was found, and the Kappa value was 0.828. A statistically significant difference was observed for the volume mismatch sign between the paramedial bithalamic tumor and the non-tumorous groups (χ^2^ = 35.465, *P* < 0.001). The presence of volume mismatch sign in paramedial bithalamic illnesses predicted the presence of tumors with a sensitivity and specificity of 69.2% and 90.9%, respectively, and PPV and NPV were 90.0% and 71.4%.

**Conclusion:**

Volume mismatch sign may indicate tumors in paramedian bithalamic diseases.

## Introduction

The thalami are paired central gray matter structures deeply embedded in the brain hemispheres. They are associated with motor, autonomic, sensory, limbic, and endocrine functions. In the resting state, their metabolic requirement exceeds that of the cerebral cortex. The thalamus receives subcortical sensory and motor input and projects to both the cortex and the striatum. Thalamic lesions can cause chronic pain, sensory loss, amnesia, dystonia, and other disorders (1).

Differential diagnosis of bilateral thalamic lesions is broad and includes genetic, vascular, neoplastic, metabolic, and inflammatory illnesses. The etiology of bilateral thalamus diseases directly determines the further management of the patients. It is important but complicated for doctors to distinguish between them.

Magnetic resonance imaging (MRI) is widely used in thalamic illnesses due to its superior soft tissue resolution compared to computed tomography (CT) although it is less precise (2) in detecting calcification than CT.

Several articles reported many typical imaging findings for thalamic lesions. Lysosomal storage disorders (LSDs) often affect thalami, and their imaging manifestations are hyperdensity on CT images, high-signal intensity on T1-weighted imaging (T1WI), and hypointensity on T2-weighted imaging (T2WI) (3–6). The characteristic imaging feature of Fabry disease is the “pulvinar sign”—T1 hyperintensity of the pulvinar thalamus (7). Alcoholic Wernicke's encephalopathy (WE) often involves symmetrically the medial thalami, periaqueductal gray matter, quadrigeminal plate, and mammillary bodies (8). The radiologic appearance of metronidazole-induced encephalopathy may mimic that of WE, but the bilateral cerebellar dentate nuclei are its characteristic location (9, 10). Acute hyperammonemic encephalopathy (AHE) symmetrically involves the bilateral cortex of the insula and cingulate gyrus, usually accompanied by thalamic involvement, with the basal ganglia and occipital cortex spared (11, 12). Both acute necrotizing encephalopathy (ANE) and deep cerebral venous thrombosis (DCVT) demonstrate symmetric hemorrhage of the bithalami. However, in addition to the thalami, ANE often affects the brainstem and sub-insular areas, as well as the cerebellum and basal ganglia (13, 14). Both DCVT and dural arteriovenous fistula (AVF) appear as bilateral thalamus abnormalities. The “midbrain V sign” with or without water diffusion restriction is the classic radiologic finding of artery of Percheron (AOP) infarction (15).

Most experts that studied thalamic lesions have found some neuroradiologic characteristics helpful, including, hyperdensity on CT images, high- and low-signal intensity on T1WI and T2WI, hemorrhage, mass effect, enhancement, and certain specific locations (3–15). Although we divided the thalamus into four parts, paramedial, lateral, anterior, and posterior areas, respectively, these arterial territories were only used in diagnosing ischemic infarctions. Other thalamic illnesses may have their preferred subregions and can be identified by these clues. Our study focused on the paramedial area as illnesses of these sub-divisions are more commonly encountered and less difficult to differentiate compared to traditional thalamic diseases previously studied. The aim of this study was to explore the potential application value of volume mismatch sign in recognizing paramedial bilateral thalamic tumors among other illnesses.

## Methods

### Participants

**Figure 1:**
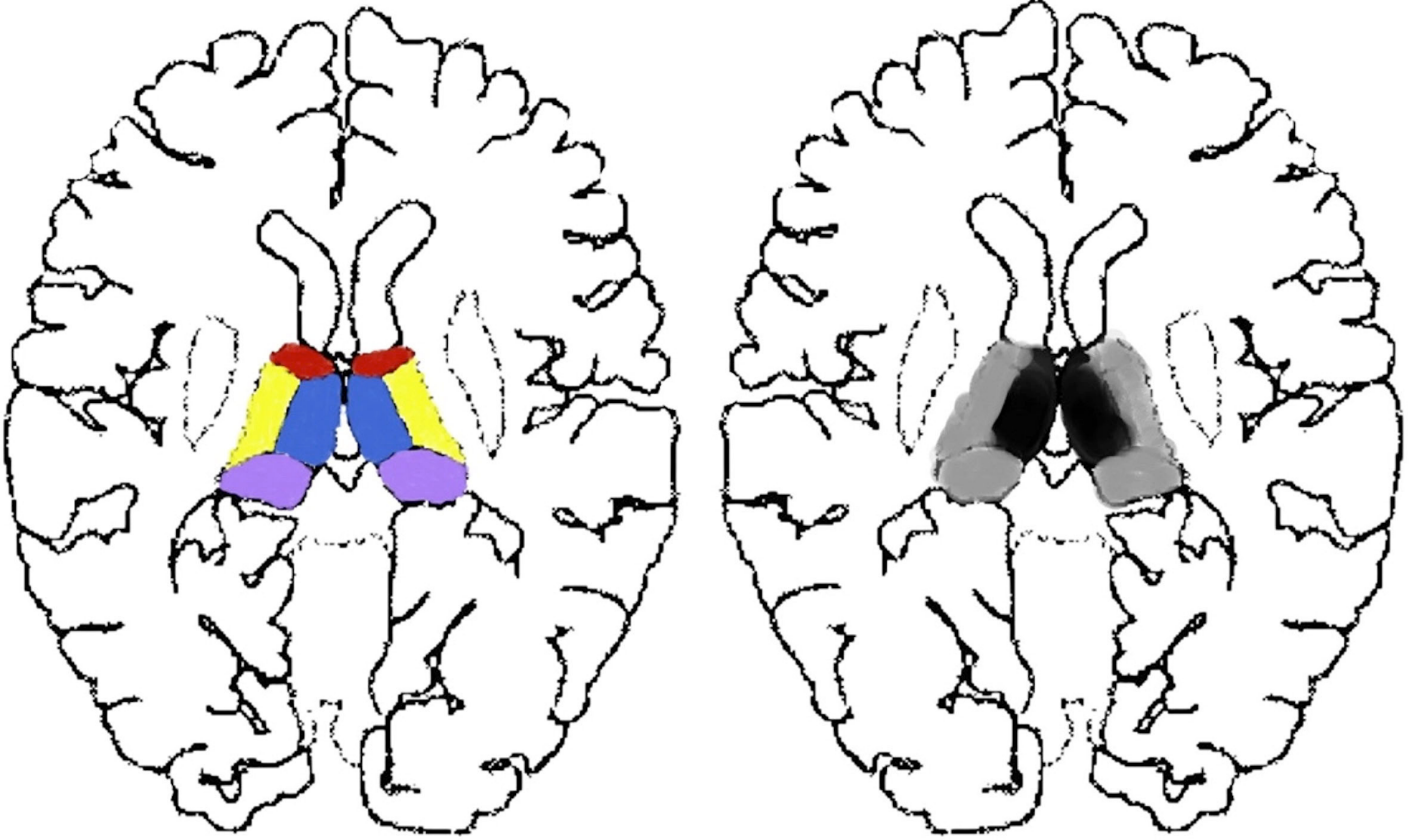
Thalamus segmentation and the exact sub-divisions studied. Each side of the thalamus is categorized into four parts, namely, the paramedial (blue), anterior (red), lateral (yellow), and pulvinar (purple) sub-divisions. We studied lesions of focal (black) and widespread (black and gray) thalamus, consisting of at least that of the paramedian (black) parts.

We performed a retrospective analysis of patients clinically diagnosed with bilateral paramedial illnesses (Figure 1), some of whom may also have had midbrain disease, using MRI between March 2012 and October 2022 in Beijing Tiantan Hospital. The etiology of these recruited subjects could not be identified on T1WI or T2WI.

**Figure 2:**
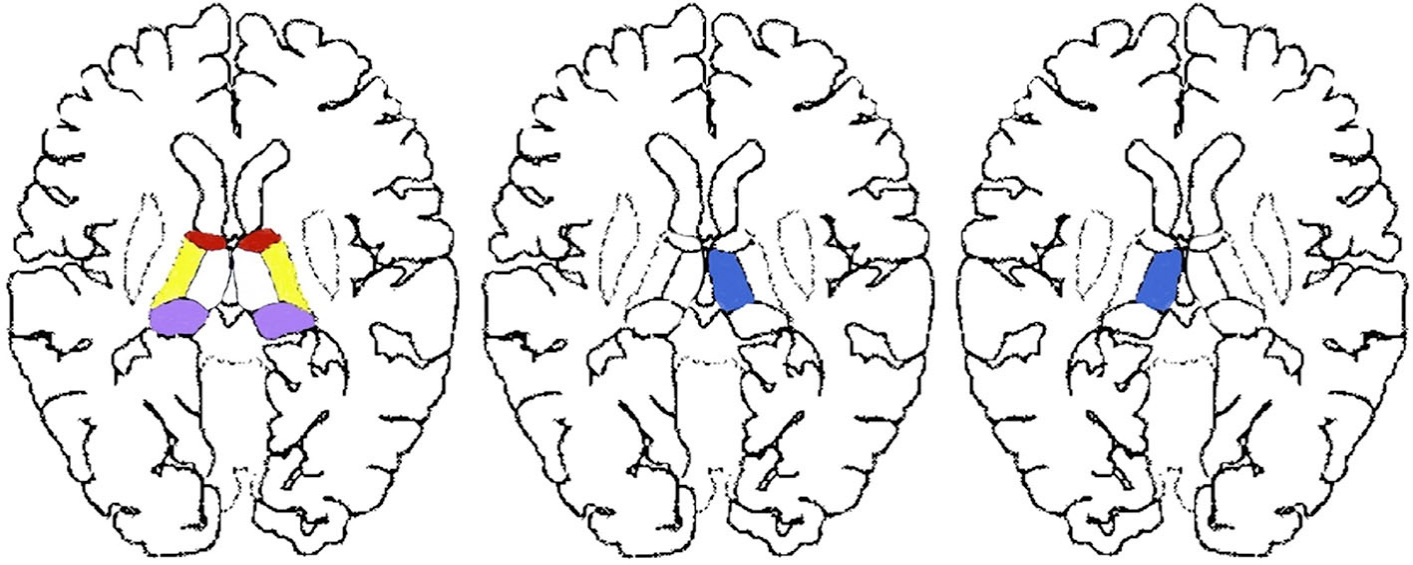
Excluded thalamic sub-regions of our study. Single-sided thalamic lesions located in the red, purple, yellow, or blue areas as well as paired lesions without paramedial involvement located in the paired red, purple, or yellow areas were not in the scope of our research.

The exclusion criteria for all participants were age >90 years, treatments before MRI scans, inferior image quality for further imaging evaluation, and cases with one-sided thalamic lesions (Figure 2) or with both-sided lesions of anterior, lateral, or pulvinar sub-regions (Figure 2).

### Imaging protocols and data reconstruction

T1WI/T1 fluid-attenuated inversion recovery (T1-FLAIR), T2WI/PROPELLER T2WI (Prop T2WI), T2-FLAIR, postcontrast T1WI, and diffusion-weighted imaging (DWI) were performed using either a 1.5 T (GE SIGNA Explorer) or a 3.0 T MRI system (GE Discovery, Siemens Verio, Philips Ingenia CX) with an eight-channel head coil. T1WI, T2WI/Prop T2WI, and FLAIR were performed in almost all patients, but DWI and postcontrast T1WI were used for some patients.

The standard MRI protocol parameters were as follows: 2D acquisition type, 100°–150° flip angle, 24 × 24 cm^2^ field of view, 512 × 512 acquisition matrix, and 5/5.5 mm or 6/6.5mm section thickness/spacing between slices.

The other parameters of these sequences are as follows: T1WI: repetition time (TR) = 6.43 ms; echo time (TE) = 2.97 ms; T1-FLAIR:TR = 1,900–2,275.2 ms, TE = 8.6–29.06 ms, inversion time (TI) = 787–950 ms, scanning time (ST) = 59–86 s. T2WI:TR = 4,500–4,744 ms, TE = 85.82–110.22 ms, ST = 52–150 s. Prop T2WI:TR = 7,385.03 ms, TE = 105.49 ms, ST = 53 s. T2-FLAIR: TR = 7,500–8,000 ms, TE = 85–91 ms, TI = 2,000–2,415 ms, ST = 75–111 s. DWI: TR = 2,200–4,200 ms, TE = 58–98 ms, ST = 30–53 s.

Apparent diffusion coefficient images were reconstructed from the data acquired with the DWI sequence by using in-house software.

### Radiological and clinical assessment

Radiological and clinical parameters, including age, gender distribution, non-specific symptoms, neurologic deficits, signal intensity heterogeneity, water diffusion restriction, larger left or right-sided lesion, the involvement of midbrain, T1 contrast enhancement, and mass effect/hydrocephalus were documented in the medical imaging diagnostic report and medical record.

The preoperative T2WI images of all participants were assessed for volume mismatch. Lesions were independently evaluated by two neuroradiologists (with more than 5 years of experience) who did not participate in lesion recruitment and were blinded to their nature.

**Figure 3:**
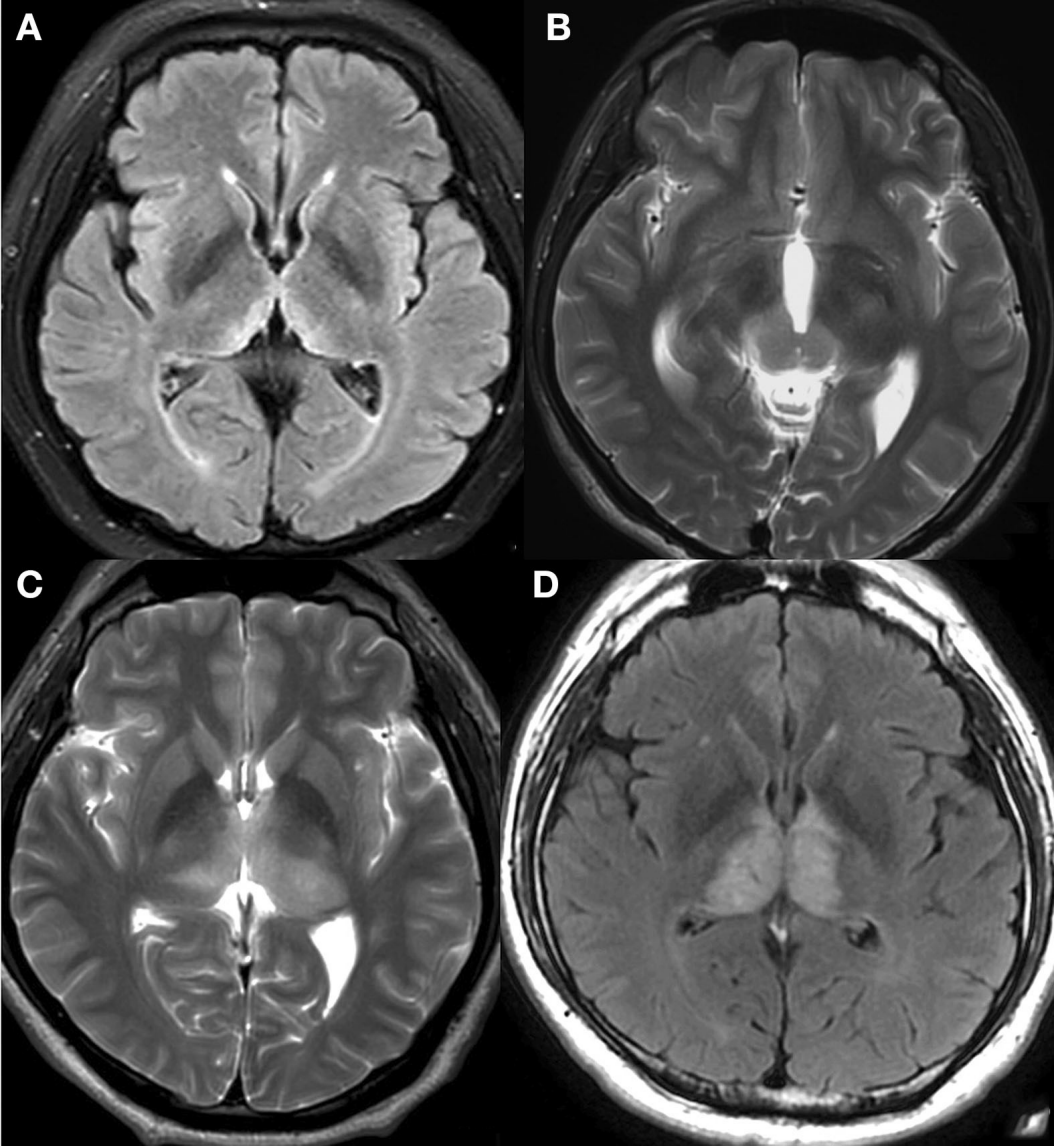
Negative and positive volume mismatch signs. **(A)** A 46-year-old woman with mental depression for 3 months, memory loss for more than 1 month, limb numbness and weakness for half a month. T2-FLAIR revealed hyperintensity on paramedial bithalami with negative volume mismatch. Observers predicted that the lesions might not be tumorous, and this prediction was later confirmed when the patient developed WE. **(B)** A 22-year-old man with no specific symptoms. T2WI revealed high signal intensity on medial parts of both the bilateral thalamus and the cerebral peduncle with positive volume mismatch. Germinoma was pathologically confirmed and the observers' prediction was correct. **(C)** A 43-year-old man with right limb numbness for more than 3 years, and aggravated limb numbness with diplopia for 2 months. T2WI showed heterogeneous high signal intensity on bithalamus with positive volume mismatch. Anaplastic astrocytoma (AA) was pathologically confirmed, and the observers' prediction was proven correct. **(D)** A 41-year-old man with aggravated memory loss for more than 2 months. T2-FLAIR revealed abnormalities of both thalami without volume mismatch sign. MRI venography showed a complete absence of flow within the straight sinus, confirming DCVT. The observers' prediction was correct.

Positive (>1.5) and negative (≤1.5) volume mismatch signs were categorized according to the volume ratio (Figures 3A–D). The volume ratio was calculated by the ratio of the larger-sided lesion volume to the smaller-sided lesion volume. The volume of each lesion was calculated by multiplying the anteroposterior diameter, the left-right diameter, and the height of the lesion, and then dividing by 2. The height of the lesion was either measured on coronal images or the sum of every layer thickness and interval. The diameters were measured using inhouse software. MRIcron software was only used when the two readers disagreed with each other on the evaluation of the volume mismatch sign. It automatically calculates the exact volume of the lesion after sketching its outline at each slice.

### Statistical analysis

Analyses were conducted using SPSS Statistics version 26 (IBM Corp., Armonk, New York, USA).

All measurements are expressed as mean ± standard deviation. Cohen's kappa for the reproducibility of two readers' assessment on volume mismatch sign on MRI T2WI or T2-FLAIR was calculated. The chi-square (χ^2^) test and the Student's *t*-test were used to analyze the differences in volume mismatch sign as well as the clinical and other neuroradiological parameters between the paramedian bithalamic tumor group and the non-tumorous group. The positive predictive values (PPV), negative predictive values (NPV), sensitivity, and specificity of volume mismatch sign in identifying tumors in paramedial bithalamus diseases were calculated. A two-sided *p* < 0.05 was considered to be statistically significant.

## Results

### Demographic, radiological, and clinical characteristics

Ninety-six patients, i.e., 44 cases with tumors and 52 with non-neoplastic diseases, were enrolled in this study.

The underlying diseases were as follows: germinoma (*n* = 13), astrocytoma (*n* = 3), AA (*n* = 3), glioblastoma (*n* = 2), diffuse midline glioma (*n* = 6), diffuse large b-cell lymphoma (*n* = 2), metastatic tumor (*n* = 1), pinealoblastoma (*n* = 1), gemistocytic astrocytoma (*n* = 1), neuronal and mixed neuronal-glial tumor (*n* = 2), rosette-forming glioneuronal tumor (*n* = 1), growing bigger after 3 months (*n* = 1) and not becoming smaller after 1 ~ 3 years (*n* = 8) and cerebral vascular diseases (*n* = 2), inflammatory disease (*n* = 15), arterial infarction (*n* = 14), venous infarction or DCVT or poor venous drainage (*n* = 10), Wilson's disease (*n* = 2), WE (*n* = 2), AVF (*n* = 1), venous anomaly (VA) (*n* = 3), and cavernous malformation (CM) accompanied by VA (*n* = 3), respectively.

Paramedial bithalamic tumors are statistically demonstrated as having more mass effect/hydrocephalus (χ^2^ = 47.856, *P* < 0.001) and more midbrain involvement (χ^2^ = 7.672, *P* = 0.006) than non-tumors (Table 1). In addition, non-tumor patients were statistically older (T =-4.203, *P* < 0.001) and presented more neurologic deficits (χ^2^ =7.680, *P* = 0.006) than tumor patients (Table 1).

**Table 1:** Demographic, radiological, and clinical information of tumor and non-neoplastic diseases in bilateral midline thalamus.

**Bilateral midline thalamus diseases**	**Tumor group (*n =* 44)**	**Non-tumor group (*n =* 52)**	**Testing statistic**	***P-*value**
Age, year ± SD	30.09 ± 20.421	47.62 ± 20.298	−4.203	<0.001^#^
Sex, male/female	27/17	36/16	0.654	0.419
Non-specific symptoms, yes/no/not obtained	28/12/4	32/8/12	1.067	0.302
Neurologic deficits, yes/no/not obtained	19/21/4	31/9/12	7.680	0.006^#^
Signal intensity heterogeneity, yes/no	28/16	26/26	1.801	0.180
Diffusion restriction, yes/no/not obtained	16/19/9	9/28/15	3.631	0.057
Larger left-sided lesion, yes/no	21/23	30/22	0.950	0.330
Midbrain involvement, yes/no	25/19	15/37	7.672	0.006^#^
Contrast enhancement, yes/no/not obtained	30/14/0	10/12/30	3.173	0.075
Mass effect/hydrocephalus, yes/no	30/14	1/51	47.856	<0.001^#^

^*^Data are mean ± standard deviation. ^#^P < 0.05 was considered statistically significant. P-values were two-tailed.

There were no statistical differences between bilateral midline thalamus tumor and non-neoplastic groups for the following parameters: sex distribution, non-specific symptoms, signal intensity heterogeneity, water diffusion restriction, larger left or right-sided lesion, and T1 contrast enhancement (Table 1).

### Interobserver agreement

Interobserver agreement of the volume mismatch sign of medial bilateral thalamus diseases was good (*K* = 0.828).

### PPV, NPV, sensitivity, and specificity

Thirty-six of fifty-two positive volume mismatch cases suffered from neoplastic diseases of the bilateral paramedian thalamus, resulting in a PPV of 69.2%. Forty out of the forty-four negative volume mismatch participants had non-tumorous illnesses, yielding an NPV of 90.9%. The sensitivity and specificity of volume mismatch sign in identifying tumors among paramedial bithalamic diseases were 90.0% (36 of 40 cases) and 71.4% (40 of 56 patients), respectively. These results are presented in Table 2.

**Table 2:** Relationships of volume mismatch signs between the tumor and non-tumor groups.

**Volume mismatch sign**	**Paramedial bithalamic diseases**		**χ^2^**	***P-*value**
	**Tumor**	**Non-tumor**		
Negative	4	40	35.465	<0.001^*^
Positive	36	16		

^*^P <0.05 was considered statistically significant. P-values were two-tailed.

## Discussion

The aim of this study was to find new imaging features for bilateral thalamus diseases aside from mass effect, specific locations, and signal intensity reported in previous studies and to evaluate their diagnostic value in distinguishing paramedian bilateral thalamus tumors from non-tumors.

As we know, the etiology of thalamus lesions is directly associated with further management (16).

### Unilateral and bilateral thalamus, basal ganglia, and brain stem lesions—variable arrangements and hard-to-make decisions

Many experts have studied thalamic lesions but without partitioning the thalamus. They focused on the interrelation of the thalamus and other regions of the brain instead. Different arrangements of the thalamus, basal ganglia, and even brain stem lesions result in different diseases (17–19). In addition to the thalamus, various conditions such as toluene toxicity/solvent abuse, LSDs, AHE, influenza A-associated encephalitis, hypoxic-ischemic encephalopathy (HIE) (20), and tick-borne encephalitis (21, 22) can involve the basal ganglia, brain stem, or cerebral white matter to varying extents.

There are six main types of thalamic diseases: metabolic, toxic, genetic, inflammatory/infectious, vascular, and tumorous, and their manifestation can vary in over 40 different ways. Therefore, it is complicated for clinicians and radiologists to handle such a complex array of diseases.

### Bilateral thalami illnesses of the paramedian parts are more commonly seen than that of the other thalamic sub-regions

Thalami is commonly divided into four parts according to the blood supply, namely, paramedial, anterior, inferolateral, and posterior lateral areas (23). Experts have reported that paramedial bilateral thalamus lesions with midbrain involvement were more commonly seen than lesions in other parts. Both the singular blood supply and venous drainage (*via* the Galen vein that drains venous blood from the bilateral thalami) (23) contribute to the high incidence of the paramedian and widespread bithalamic lesions among thalamic disorders.

Although many diseases appear as widespread abnormal bilateral thalami, they may be predisposed to certain subregions. HIE typically affects both the thalamus and the gray matter of the brain. If only the bithalami are involved, it may predominantly affect the lateral portions (24). It has been reported that hemolytic uremic syndrome often involves the lateral parts of the thalami on both sides (19, 25). Different subregions of the thalami may link to different illnesses.

### Paramedian bithalami illnesses—relatively less challenging to make differential diagnoses

Distinguishing tumors from non-tumors and differential diagnosis of illnesses involving the paramedial thalami are generally straightforward tasks for radiologists and clinicians.

We believe that the paramedial region is a critical area for the differential diagnosis of thalamic diseases for several reasons. First, special vessel anatomy (23) makes paramedial lesions and lesions that affect the entirety of the bithalami are the most common. Second, bilateral thalami and pedunculus cerebri are naturally paired and directly connected to each other by the interthalamic, anterior, posterior, habenular commissure, and midbrain.

### Possible reasons for positive volume mismatch of paramedial bithalamic tumors

Third, paramedian bithalamus vascular diseases, including “top of the basilar” infarction, AOP infarction (26), and straight sinus thrombosis, present symmetrical appearances due to symmetrical vessel architecture and equal probabilities of embolism and atherosclerosis. Fourth, tumors, including glioma and germinoma, tend to originate from one spot and invade the other side from the five connection structures we mentioned previously. Therefore, bilateral paramedial thalamic tumors tend to be asymmetrical. Finally, inflammatory, toxic, and metabolic illnesses of the thalamus are related to blood supply or genetic factors and are commonly systemic, randomly distributed, and symmetrical (18). In this study, there were 44 cerebral tumors and 52 non-tumors, and the tumors indeed tended to be asymmetrical.

In this study, three cases were identified with bad venous drainage of bilateral thalami induced by giant tumors located elsewhere, which have not been reported before. Two of them were compressed by angioreticuloma of the left cerebellar, and one of them was induced by meningioma of the anterior cranial fossa. Additionally, six patients had bilateral thalamic edema and swelling due to VA, three of whom were accompanied by CM, in our study. These findings are consistent with previous reports (27).

Although our hospital is specialized in neurology and neurosurgery, some kinds of thalamic diseases were absent in our study, including H1N1 infection-associated ANE (28, 29), multiple sclerosis (30), atezolizumab-induced encephalitis (31), West Nile encephalitis (20), Reye syndrome (32), and acute disseminated encephalomyelitis (33), which may be due to their low prevalence, atypical appearance, or selection bias in the respective studies.

## Limitations

The limitations of our study include selection bias because of its monocentric and retrospective nature. As our study was a retrospective study, the thickness and layer spacing of MRI scans may not be exactly the same, which could result in measurement errors. Additionally, the number of patients with bilateral medial thalamus lesions that were not included in our approach cannot be provided as they might conduct CT scans respecting MRI contraindications.

## Conclusion

Volume mismatch may help clinicians identify tumors in bithalamic paramedial diseases.

## Data availability statement

The original contributions presented in the study are included in the article/supplementary material, further inquiries can be directed to the corresponding author.

## Author contributions

PG conceived and designed the research strategy and participated in the writing of the manuscript. LS collected clinical materials, wrote the manuscript text, and contributed to the analysis of the data. All authors contributed to this manuscript and approved the submitted version of the manuscript.
